# Socio-economic and geographical inequalities in adolescent fertility rate in Ghana, 1993–2014

**DOI:** 10.1186/s13690-021-00644-x

**Published:** 2021-07-06

**Authors:** Bright Opoku Ahinkorah, Eugene Budu, Henry Ofori Duah, Joshua Okyere, Abdul-Aziz Seidu

**Affiliations:** 1 School of Public Health, Faculty of Health, University of Technology Sydney, Ultimo, Australia; 2 Department of Population and Health, University of Cape Coast, Cape Coast, Ghana; 3 Research Department, FOCOS Orthopaedic Hospital, Accra, Ghana; 4 College of Public Health, Medical and Veterinary Sciences, James Cook University, Townsville, Queensland Australia

**Keywords:** Adolescent fertility, Inequality, Ghana, Demographic and Health Surveys, Global health

## Abstract

**Background:**

Despite public health interventions to control adolescent fertility, it remains high in sub-Saharan Africa. Ghana is one of the countries in sub-Saharan Africa with the highest adolescent fertility rates. We examined the trends and socio-economic and geographical patterns of disparities in adolescent fertility in Ghana from 1993 to 2014.

**Methods:**

Using the World Health Organization’s (WHO) Health Equity Assessment Toolkit (HEAT) software, data from the 1993–2014 Ghana Demographic and Health surveys were analyzed. First, we disaggregated adolescent fertility rates (AFR) by four equity stratifiers: wealth index, education, residence and region. Second, we measured the inequality through summary measures, namely Difference (D), Population Attributable Risk (PAR), Ratio (R) and Population Attributable Fraction (PAF). A 95 % confidence interval was constructed for point estimates to measure statistical significance.

**Results:**

We observed substantial absolute and relative wealth-driven inequality in AFR (PAR=-47.18, 95 % CI; -49.24, -45.13) and (PAF= -64.39, 95 % CI; -67.19, -61.59) respectively in favour of the economically advantaged subpopulations. We found significant absolute (D = 69.56, 95 % CI; 33.85, 105.27) and relative (R = 3.67, 95 % CI; 0.95, 6.39) education-based inequality in AFR, with higher burden of AFR among disadvantaged subpopulations (no formal education). The Ratio measure (R = 2.00, 95 % CI; 1.53, 2.47) indicates huge relative pro-urban disparities in AFR with over time increasing pattern. Our results also show absolute (D, PAR) and relative (R, PAF) inequality in AFR across subnational region, between 2003 and 2014. For example, in the 2014 survey, the PAR measure (D=-28.22, 95 % CI; -30.58, -25.86) and the PAF measure (PAF=-38.51, 95 % CI; -41.73, -35.29) indicate substantial absolute and relative regional inequality.

**Conclusions:**

This study has indicated the existence of inequality in adolescent fertility rate in Ghana, with higher ferlitiy rates among adolescent girls who are poor, uneducated, rural residents and those living in regions such as Northern, Brong Ahafo, and Central region, with increasing disparity over the time period of the study. There is the need for policy interventions that target adolescent girls residing in the rural areas and those in the low socioeconomic subgroups to enable the country to avert the high maternal/newborn morbidity and mortality usually associated with adolescent childbearing.

## Background

Globally, demographic change has been a public health issue over the past few years [1]. Adolescent fertility has fallen considerably since 2004 [1, 2]. However, inter-country, subnational and sub population disparities show that large numbers of young people do not have access to means of controlling their fertility with important implications for their health. Adolescent fertility is considered high in low-and middle-income countries, with higher rates in sub-Saharan African countries [3].

In sub-Saharan Africa, adolescent fertility continues to be high despite the advancement of sexual and reproductive healthcare services to control fertility rate [4]. Adolescent fertility is measured by the adolescent fertility rate [AFR], which is the annual number of births to women aged 15 to 19 years per 1,000 women in that age group [3]. The Sustainable Development Goal (SDG) 3.7 recognizes the interdependence between ensuring universal access to sexual and reproductive healthcare services and other developmental goals, including ending poverty in all its forms, due to its association with teenage marriages, pregnancies and births [1].

Despite public health interventions to control adolescent fertility, it remains high in sub-Saharan Africa unlike other developing regions where it has significantly declined [5]. The AFR varies among countries within the sub-Saharan Africa region. Interestingly, trends in Ghana’s AFR showed an uneven trend. Meanwhile, the 2019 World Fertility policy document illustrated the variations in the trends in adolescent fertility across countries to be associated with trends in the growth in national wealth, income inequalities and educational expenditures [6]. According to Ghana Demographic and Health Surveys (GDHS), from 1993 to 2014, the country recorded an AFR of 118.73 births per 1000 adolescent girls in 1993, reduced to 90.29 births per 1000 adolescent girls in 1998, declined again to 73.83 births per 1000 adolescent girls in 2003, further declined to 69.69 births per 1000 adolescent girls in 2008 and with the recent survey in 2014, the rate rose to 76 births per 1000 adolescent girls. Evidence from the 2014 GDHS showed that there has been an increase in the AFR over the past six years with little understanding of the reason for the increase [7, 8]. Notwithstanding, no study has investigated the trends in the inequalities of AFR in Ghana and its magnitudes. Therefore, this study sought to critically examine the trends and socio-economic and geographical patterns in adolescent fertility in Ghana from 1993 to 2014. Findings from this study will help to formulate useful interventions and strategies in controlling AFR in Ghana.

## Methods

### Study area

The area for the study is the Republic of Ghana. The country is among the countries in the West African sub-region and has a total land area of 238,533km^2^ [7]. The population of the country in 1960, 1970, 1984, 2000 and 2010 were 6,726,815; 8,559,313; 12,296,081; 18,912,079 and 24,658,823 respectively [7]. At the time of the surveys, the country was divided into ten regions. These regions were Western Region, Central Region, Greater Accra Region, Volta Region, Eastern Region, Ashanti Region, Brong Ahafo Region, Northern Region, Upper East Region and Upper West Region. Currently, the country has 16 regions, namely Oti Region, Brong Ahafo Region, Bono East, Ahafo Region, North East Region, Savannah Region, Western North Region, Western Region, Greater Accra Region, Central Region, Eastern region, Upper East Region, Upper West Region, Volta Region, Northern Region and Ashanti Region. In terms of geographical distribution, about 51 % of Ghana’s population is urbanised whiles 49 % of the population live in rural areas. In relation to ethnicity, the distribution are as follows; Akan (47.5 %), Mole Dagbani (16.6 %), Ga-Adangbe (7.4 %), Gruma (5.7 %), Guan (3.7 %), Grusi (2.5 %) and ‘other’ (Mande, Hausa and other ethnic groups) (16.6). In terms of religion, the majority of Ghanaians (71.2 %) are Christians (Catholic, Protestant, Pentecostal/Charismatic and other Christian) followed by Muslims (17.6 %), Traditionalist (5.2 %), No Religion (5.3 %) and 0.8 % of the population belonged to ‘other’ religion [7].

### Data source and study population

In this study, data from five rounds (1993, 1998, 2003, 2008, and 2014) of the GDHS was used. The DHS is part of the surveys carried out in low-and middle-income countries every five years under the MEASURE DHS program. Each of the surveys focuses on collecting data on women, children, men and households. In terms of the surveys for women, some of the key issues considered are fertility, family planning and utilization of maternal health services such as antenatal care visits. The sampling approach employed in the DHS is a two-stage stratified sampling. The first stage was the selection of clusters across urban and rural locations from the entire nation, which constituted the enumeration areas (EAs) for the study. The next stage was the selection of households from the predefined clusters. Details of the methodologies employed in the various rounds of the surveys can be found in the final reports of the GDHS [8]. In this study, adolescents aged 15–19 years in the respective GDHS rounds were included.

### Measures of inequality

The inequality variable measured in this study is AFR. It is measured as the proportion of births per 1000 women aged 15–19. We disaggregated AFR by four equity stratifiers: economic status, education, subnational region and place of residence. We approximated economic status through a composite variable known as wealth index. In the DHS, wealth index is computed using different household ownerships and characteristics following Principal Component Analysis (PCA) technique [9]. Wealth index had five categories: poorest, poorer, middle, richer and richest. Educational status of the woman was classified as no-education, primary, secondary/higher and place of residence as urban vs. rural. Subnational regions were in the then ten regions of Ghana.

### Statistical analyses

We used the 2019 updated version of the World Health Organization’s (WHO) Health Equity Assessment Toolkit (HEAT) software [10], for analysing the socio-economic and geographical inequalities associated with AFR. We carried out the data analyses following two steps. First, we disaggregated AFR by four equity stratifiers; economic status, education status, place of residence and subnational region. The purpose was to present the estimates of AFR across the various categories of the equity stratifiers. Thereafter, we calculated summary measures and used a combination of absolute and relative inequality summary measures. These were Difference (D), Population Attributable Risk (PAR), Population Attributable Fraction (PAF) and Ratio (R). The first two (D and PAR) are absolute inequality measures while the other two (PAF and R) are relative measures of inequality. Moreover, D and R estimates are simple measures, PAR and PAF are complex measures. These measures were calculated for each of the four equity stratifiers. We chose these summary measures because scientific evidence shows the importance of both absolute and relative summary measures in a single health inequality study [11, 12]. The difference between complex and absolute measures is that whiles complex measures account for size of categories of a sub-population, simple measures do not but they make it easy for one to interpret and understand the results [11, 12]. Hence, combining both relative and absolute measures in a single inequality analysis helps to provide a more comprehensive analysis. Detailed procedure and calculation of the summary measures are available in the HEAT software technical notes [10] and in the WHO handbook on health inequality monitoring [11].

The PAF and PAR assume positive values for favourable health intervention indicators and negative values for adverse health outcome indicators like AFR. A value of zero shows absence of inequality, and the greater absolute value of PAF and PAR, the higher level of inequality. The PAR estimate is calculated as the difference between the subgroup with the lowest estimate and the national average of the indicator for adverse outcome indicators. For ordered dimensions like wealth and education, PAR is the difference between the most-advantaged subgroup and the national average, regardless of the indicator type. The PAF estimate is calculated by dividing the PAR by the national average “µ” and multiplying the fraction by 100: PAF = [PAR / µ] * 100. For binary dimensions like residence, “D” is calculated as the difference between the subgroup with the highest estimate (rural) and the subgroup with the lowest estimate (urban), regardless of the indicator type. For ordered dimensions like wealth and education, it is the difference between the most-disadvantaged subgroup and the most advantaged subgroup. For binary dimensions like residence, “R” is calculated as the ratio between the subgroup with the highest estimate (rural) and the subgroup with the lowest estimate (urban), regardless of the indicator type. For ordered dimensions like wealth and education, it is the ratio between the most-disadvantaged subgroup and the most advantaged subgroup. In the absence of inequality, D and R become zero and one, respectively. Point estimates were calculated and presented with corresponding 95 % Confidence Intervals. To examine whether AFR shows statistically significant disparities across the sub-groups of each equity stratifier, and to determine whether or not the inequality changed with time, we computed 95 % Confidence Intervals (CI) around point estimates of each measure for each survey. For all inequality measures other than R, the lower and upper bounds of the CI must not include zero to interpret that inequality exists. For R, the interval should not include one. We assessed the trend of inequality for each summary measure by referring to the CI for the different survey years. When the CIs do not overlap, it implies that there is statistically significant difference between the two CIs. If the CIs overlap, then no inequality exists.

## Results

**Fig. 1:**
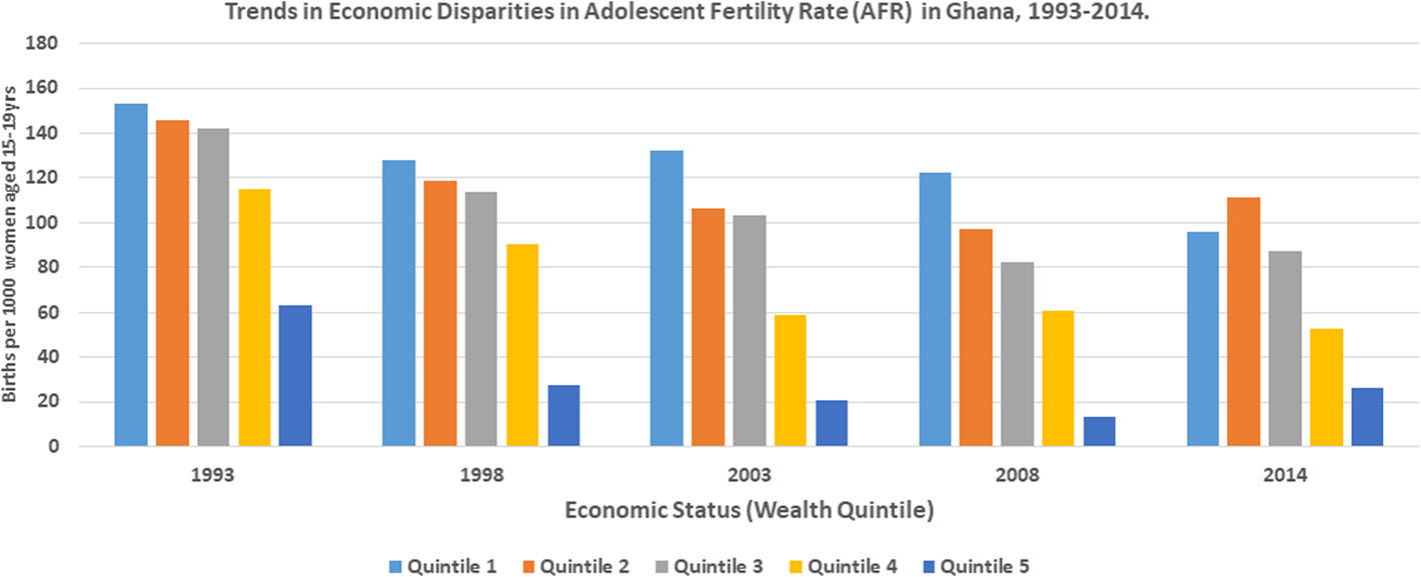
Trends in the Economic Disparities in Adolescent Fertility Rate in Ghana ,1993–2014

**Fig. 2:**
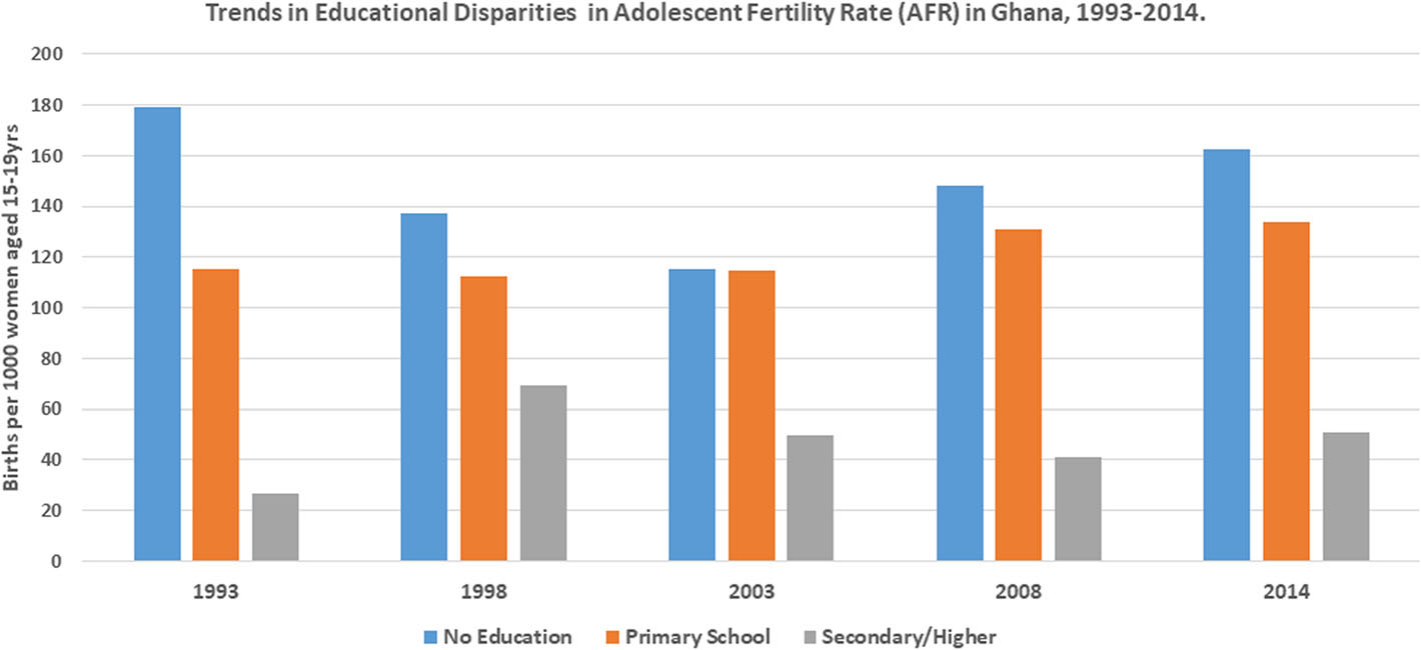
Trends in the Educational Disparities in Adolescent Fertility Rate in Ghana ,1993–2014

**Fig. 3:**
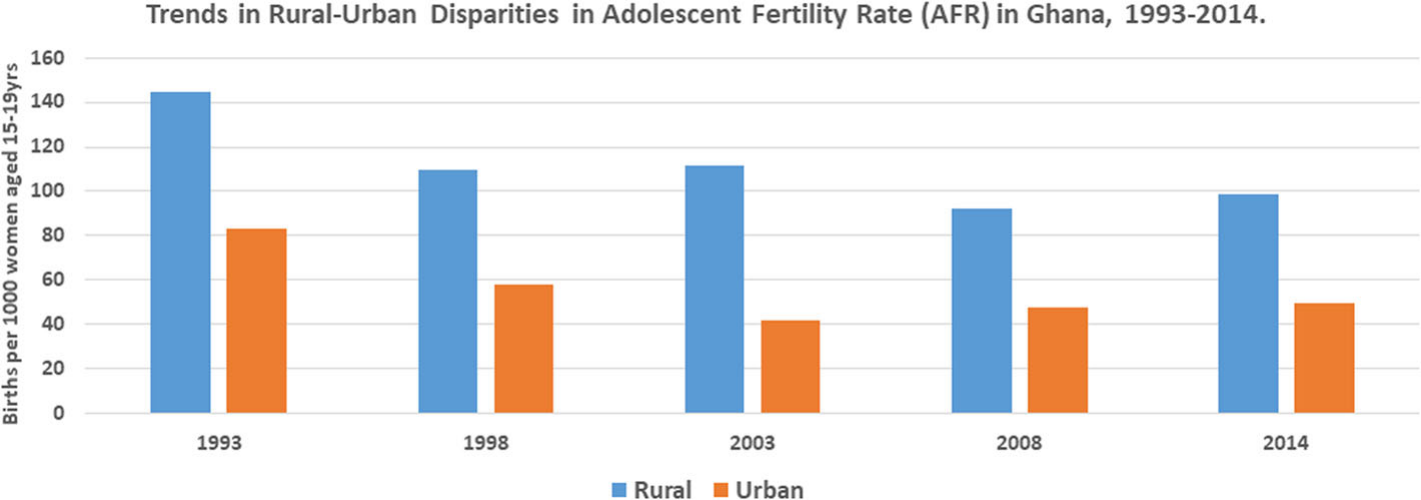
Trends in the Rural-urban Disparities in Adolescent Fertility Rate in Ghana ,1993–2014

Table 1 shows the trends and disparities in AFR across socio-economic, urban-rural and subnational populations in Ghana from 1993 to 2014. The results revealed disproportionately higher AFR among the disadvantaged subgroups. The magnitude of AFR was different across wealth quintiles with higher concentration among those in the poorest, compared to the richest wealth quintile. In 2014, for instance, while AFR was approximately 96 per 1000 adolescents in the poorest wealth quintile, it was 26 per 1000 adolescents in the richest wealth quintile. In the same survey there was disparity in terms of education. Specifically, whiles AFR was almost 163 per 1000 adolescents with no formal education, it was approximately 51 per 1000 adolescents with secondary/higher education. AFR was also high among adolescents who lived in rural areas (98.55 per 1000 adolescents), compared to those who lived in urban areas (49.23 per 1000 adolescents). Still in 2014, AFR varied by sub-regions with highest AFR recorded in the Brong Ahafo region, compared to the Greater Accra region. The economic, educational and rural-urban disparities in AFR over the years have been illustrated by Figs. 1, 2 and 3, respectively.

**Table 1:** Trends in adolescent (15-19 years) fertility rate per 1000 by different inequality dimensions, 1993–2014

		1993 (118.73)		1998 (90.29)		2003 (73.83)		2008 (69.69)		2014 (73.28)
Dimension	Sample	**Rate (95% CI)**	**Sample**	**Rate (95% CI)**	**Sample**	**%**	**Sample**	**Rate (95% CI)**	**Sample**	**Rate (95% CI)**
**Economic status**										
Quintile 1 (poorest)	553	153.57 [112.60, 205.99]	839	128.18 [98.36, 165.39]	758	132.37 [100.37, 172.62]	662	122.59 [87.18, 169.71]	1352	95.65 [68.66, 131.75]
Quintile 2	775	145.63 [112.84, 185.97]	671	118.45 [88.38, 156.99]	827	106.35 [76.44, 146.12]	876	96.97 [73.78, 126.45]	1438	111.63 [85.75, 144.09]
Quintile 3	823	142.13 [108.24, 184.45]	882	113.69 [86.34, 148.30]	1028	103.46 [77.80, 136.35]	1047	82.26 [60.17, 111.50]	1726	87.10 [65.55, 114.86]
Quintile 4	1095	115.04 [88.24, 148.66]	1001	90.13 [68.85, 117.16]	1294	59.14 [44.26, 78.59]	1146	60.74 [43.07, 85.02]	1758	52.89 [35.34, 78.45]
Quintile 5 (richest)	997	63.18 [46.69, 84.97]	1129	27.28 [18.35, 40.39]	1554	20.57 [13.74, 30.70]	1092	13.10 [6.82, 25.03]	1556	26.09 [13.32, 50.49]
**Education**										
No education	972	178.92 [140.84, 224.61]	909	137.42 [104.43, 178.74]	898	115.53 [86.14, 153.28]	525	148.21 [106.03, 203.36]	626	162.71 [97.45, 259.13]
Primary school	2745	115.08 [99.46, 132.80]	784	112.16 [87.15, 143.21]	1127	114.55 [89.42, 145.60]	915	131.03 [100.58, 168.97]	1257	133.61 [106.92, 165.72]
Secondary/higher	526	26.58 [15.10, 46.39]	2830	69.09 [57.84, 82.34]	3437	49.57 [41.27, 59.43]	3384	40.93 [32.86, 50.88]	5949	51.11 [40.27, 64.67]
**Place of residence**										
Rural	2459	144.74 [130.18, 160.63]	2827	109.81 [97.57, 123.38]	2488	111.79 [98.92, 126.10]	2368	92.42 [78.71, 108.24]	3819	98.55 [88.08, 110.10]
Urban	1785	82.89 [70.98, 96.60]	1697	57.78 [45.41, 73.25]	2975	42.08 [34.27, 51.57]	2456	47.78 [38.34, 59.39]	4013	49.23 [40.05, 60.38]
**Region**										
Western	430	151.10 [117.51, 192.20]	580	104.37 [76.70, 140.49]	529	86.43 [63.79, 116.11]	393	69.46 [46.93, 101.65]	929	72.58 [54.58, 95.91]
Central	438	127.73 [103.77, 156.26]	519	90.35 [69.34, 116.93]	431	116.01 [84.11, 157.91]	430	110.16 [72.75, 163.43]	733	92.56 [62.71, 134.58]
Greater accra	634	72.56 [52.42, 99.61]	754	44.87 [27.42, 72.61]	1017	35.57 [24.01, 52.40]	898	34.76 [21.68, 55.29]	1418	45.06 [32.30, 62.54]
Volta	385	80.47 [61.73, 104.26]	479	85.13 [61.03, 117.56]	431	92.61 [62.93, 134.28]	418	74.93 [49.54, 111.81]	604	82.37 [62.94, 107.12]
Eastern	488	120.88 [94.04, 154.09]	538	102.37 [78.26, 132.84]	565	75.06 [51.58, 108.00]	458	87.38 [60.84, 123.98]	769	88.44 [63.25, 122.36]
Ashanti	696	146.45 [122.36, 174.33]	740	106.26 [83.60, 134.17]	1164	65.85 [50.23, 85.89]	1005	52.82 [37.06, 74.77]	1424	57.24 [41.02, 79.34]
Brong-Ahafo	526	121.54 [88.96, 163.89]	351	115.12 [80.68, 161.69]	522	95.81 [69.58, 130.55]	417	80.07 [59.24, 107.40]	720	103.52 [77.02, 137.76]
Northern	337	115.67 [84.30, 156.72]	212	140.96 [91.82, 210.33]	378	84.82 [56.10, 126.27]	443	107.14 [77.52, 146.28]	667	96.42 [77.82, 118.90]
Upper west	89	NA	99	NA	134	78.69 [52.02, 117.35]	225	65.48 [41.88, 100.97]	356	66.37 [44.52, 97.85]
Upper east	219	159.21 [101.21, 241.52]	248	54.64 [33.96, 86.78]	288	67.76 [44.98, 100.87]	133	74.99 [48.62, 113.95]	207	60.85 [41.03, 89.36]

### Magnitude of AFR based on the summary measures

We observed substantial absolute and relative wealth-driven inequality in AFR from 1993 to 2014 both by simple (D, R) and complex (PAF, PAR) measures. For instance, in the 2014 survey, the PAR measure (PAR=-47.18, 95 % CI; -49.24, -45.13) and the PAF measure (PAF= -64.39, 95 % CI; -67.19, -61.59) respectively, showed significant absolute and relative economic-related inequality in favour of the economically advantaged subpopulations. In terms of educational level, we found significant absolute (D = 69.56, 95 % CI; 33.85, 105.27) and relative (R = 3.67, 95 % CI; 0.95, 6.39) education-based inequality in AFR, with higher burden of AFR among disadvantaged subpopulations (no formal education). The pattern was replicated in all the survey rounds based on simple measures (D, R), while complex measures (PAR, PAF) suggested persistent disparities to the disadvantage of adolescent girls with no formal education.

We also found absolute and relative urban-rural inequality in AFR from 1993 to 2014 both by simple (D, R) and complex (PAR, PAF) measures with an increasing pattern in magnitude of disparity over the years. For instance, in 2014, the R measure (R = 2.00, 95 % CI; 1.53, 2.47) indicated high burden of AFR to the disadvantage of rural girls with an increasing pattern during the period under study. Our finding also shows absolute (D, PAR) and relative (R, PAF) inequality in AFR across subnational region, between 2003 and 2014. For example, in 2014 survey, the PAR (D=-28.22, 95 % CI; -30.58, -25.86) and the PAF measures (PAF=-38.51, 95 % CI; -41.73, -35.29) indicated substantial absolute and relative regional inequality (Table 2).

**Table 2:** Inequality indices estimates of the factors associated with adolescent fertility rate (15–19 years), 1993–2014

	1993			1998			2003			2008			2014		
Dimension	Estimate	Lower Bound	Upper Bound	Estimate	Lower Bound	Upper Bound	Estimate	Lower Bound	Upper Bound	Estimate	Lower Bound	Upper Bound	Estimate	Lower Bound	Upper Bound
**Economic status**															
D	90.39	40.26	140.52	100.90	65.89	135.90	111.80	74.95	148.64	109.49	67.74	151.24	69.56	33.85	105.27
PAF	-46.79	-48.88	-44.70	-69.78	-72.45	-67.11	-72.14	-74.67	-69.60	-81.20	-83.98	-78.43	-64.39	-67.19	-61.59
PAR	-55.55	-58.03	-53.07	-63.01	-65.42	-60.60	-53.26	-55.13	-51.38	-56.60	-58.53	-54.66	-47.18	-49.24	-45.13
R	2.43	1.40	3.46	4.70	2.48	6.92	6.43	3.32	9.55	9.36	2.52	16.20	3.67	0.95	6.39
**Education**															
D	152.34	107.98	196.69	68.33	29.43	107.22	65.96	31.45	100.47	107.28	58.13	156.43	111.60	30.73	192.48
PAF	-77.61	-80.70	-74.52	-23.48	-24.31	-22.65	-32.86	-34.24	-31.47	-41.28	-42.76	-39.80	-30.25	-31.08	-29.42
PAR	-92.15	-95.81	-88.48	-21.20	-21.95	-20.45	-24.26	-25.28	-23.24	-28.77	-29.80	-27.74	-22.17	-22.78	-21.56
R	6.73	2.64	10.82	1.99	1.35	2.63	2.33	1.54	3.13	3.62	2.20	5.04	3.18	1.45	4.92
**Place of residence**															
D	61.85	42.00	81.69	52.03	33.16	70.91	69.71	53.66	85.76	44.64	26.60	62.69	49.32	34.40	64.23
PAF	-30.18	-30.63	-29.74	-36.01	-37.98	-34.04	-43.00	-44.74	-41.27	-31.45	-33.46	-29.43	-32.82	-34.30	-31.33
PAR	-35.83	-36.36	-35.31	-32.51	-34.30	-30.73	-31.75	-33.03	-30.47	-21.92	-23.32	-20.51	-24.05	-25.13	-22.96
R	1.75	1.42	2.07	1.90	1.39	2.41	2.66	2.03	3.29	1.93	1.41	2.46	2.00	1.53	2.47
**Region**															
D							80.43	41.33	119.54	75.40	27.87	122.93	58.46	24.88	92.04
PAF							-51.82	-55.51	-48.12	-50.12	-54.27	-45.98	-38.51	-41.73	-35.29
PAR							-38.26	-40.98	-35.53	-34.93	-37.82	-32.05	-28.22	-30.58	-25.86
R							3.26	1.63	4.90	3.17	1.21	5.13	2.30	1.29	3.31

## Discussion

AFR is a major population concern for most countries across the globe. Although there is a plethora of knowledge and literature on AFR, most of these studies have mainly involved country-level and multi-country level analysis. Consequently, the dynamics of inequalities in AFR within sub populations has been rarely explored. To that effect, we examined the trends in the disparities in AFR spanning from 1993 to 2014 in Ghana using different dimensions and measures of inequality. Findings of this study show disparity in the prevalence, absolute and relative differences in AFR within the period under study (1993–2014). It is worth nothing that AFR is considered an adverse health outcome in the Health Equity Assessment Toolkit (HEAT) analysis [12]. This implies that it is a health outcome for which public health interventions seek to ameliorate.

The study revealed that there has been significant improvement in the prevalence of AFR in Ghana from 1993 to 2014; reducing dramatically from 118.7 % to 1993 to 73.3 % in 2014. Thus, on a prima facie basis, it can be observed that Ghana has seen consistent reduction in the prevalence of AFR throughout the 16-years period, except for the period between 2008 and 2014 where the rate of adolescent fertility increased marginally from 69.7 to 73.3 %. This seemingly decreasing trend in the prevalence of AFR in Ghana over the years may be attributed to the steady increase of modern contraceptives from 1998 to 2008 [13]. Also, this may also be explained by the commitment and unwavering efforts and interventions by the government to increase modern contraceptive use among the population [14]. However, the marginal increase in AFR between 2008 and 2014 may be explained by the low use of modern contraceptives among women 15–19 years [8]. This may also be attributable to the dominance of health prioritization for other infectious diseases like HIV and non-communicable diseases to the detriment of general reproductive health [15].

Our findings also suggest that there is inequality with regards to the dimension of wealth or economic status. From the results, it can be observed that the prevalence of AFR was low among those in the richest wealth quintile compared to those in the poorest wealth quintile in 1998. Moreover, the study showed both significant absolute and relative economic related inequality in favour of the economically advantaged subpopulations. This finding corroborates previous body of knowledge that posit that AFR is low among women in the richest wealth quintile [16–18]. For instance, Osmani-Samani et al. [19] reported that inequality in the distribution of wealth contributed more to the inequality in AFR. Obviously, this is attributable to the fact that adolescents within the richest wealth quintile have the financial resources and capacity to easily afford quality [20], timely reproductive health services including access and use of modern contraceptives, comprehensive abortion care and family planning services [13]. Thus, translating into the low AFR among this sub population. On the contrary, the high AFR among adolescents within the poorest wealth quintile points to what Neal, Channon, et al. [17] describe as a recipe for compounding of vulnerability. Thus, if this disparity is left unabatted, it will perpetuate a much more vicious cycle of income inequality in the AFR of adolescents in Ghana. Irrespective of the sub population, there was an overall pattern of decline in the prevalence of AFR among the different wealth quintiles.

With respect to education, the contribution of educational inequality to AFR is seen in both the absolute (D = 69.56, 95 % CI; 33.85, 105.27) and relative inequalities (R = 3.67, 95 % CI; 0.95, 6.39). This implies that education plays a significant role in AFR in Ghana. Consistent with extant literature, the results of our study suggest that disadvantaged sub populations (no formal education) suffered a higer burden of AFR compared to thier counterparts with secondary or higher education. Our finding falls in line with the postulation by Pons-Duran, Lucas, et al. [20] and Asamoah [20] that higher educational level is a significant driver of AFR reduction as it shapes, reinforces and strenghtens the ability of women to access health care services including reproductive health services like modern contraceptives, family planning, abortion, among others. Another plausible explanation for this observation is that, adolescents girls with secondary or higher education tend to be engulfed by their involvements and responsibilities in their professional careers during their education, making it a disincentive for engage in unprotective sex leading to reduced burden of AFR in this sub-population [20, 21].

Our study also found absolute and relative urban-rural inequality in AFR from 1993 to 2014, demonstrating pro-rural disparities (R = 2.00, 95 % CI; 1.53, 2.47) in AFR with over time increasing pattern. This is an indication that Ghana has a long way to go to bridge the inequalities in the rural-urban dichotomy. This finding affirms several studies that have found that adolescents dwelling in urban areas tend to have lower AFR compared to their counterparts in the rural areas [22–24]. One possible explanation for this observation is the situation of most of the adolescent friendly health corners and facilities in the urban areas, relegating the rural dwellers to the background. Subsequently, urban dwelling adolescents have ‘unrestricted’ access to a wide range of sexual and reproductive health information, services and interventions. Hence, making it an unsurprising phenomenon to see that AFR is higher in the rural areas. Therefore, there is the need for government and interventionists to rethink and roll out intervention tailored to the needs of rural-based adolescents so as to decrease the magnitude of the pro-rural disparity in AFR.

Besides the inequalities observed among subpopulations in education, wealth quintile and place of residence, our findings revealed that there was inequality in AFR across subnational region, between 2003 and 2014. This was evident in the PAR measure (D=-28.22, 95 % CI; -30.58, -25.86) and the PAF measure (PAF=-38.51, 95 % CI; -41.73, -35.29). There is no clear explanation for the inequalities across the subnational region. However, we can speculate that it has its roots deep-seated in the dynamics of each region. Greater Accra is known to be the most urbanized region [7] in Ghana and as such, the results showing the low AFR in this region is reflective of the pro-rural disparity in the burden of AFR in Ghana.

From policy standpoint, there is the need to demystify discourse of adolescent marriage and pregnancies at the community and household level so that adolescents will be well informed about the options available to them. From the cultural standpoint, parents should be admonished to have open discussion with the female adolescent about their sexual behaviours with special emphasis on disadvantaged subgroups such as those in the rural areas and low-income households. Moreover, public health professionals in the Ghana Health Service can use the information from this study as a guide in the formulation and implementation of policies to address the socioeconomic and regional disparities in AFR in Ghana.

### Strengths and limitations

The study has several strengths. First, to the best of our knowledge, this study is the first to examine socio-economic and geographical inequalities in AFR in Ghana from 1993 to 2014. Hence, the findings can be essential in guiding both policy and future research on adolescent fertility rates in this country. Secondly, the use of both simple and complex measures of inequality contributes to the quality of our results as the limitations of each group of measure is dealt with by the strengths of the others. Thirdly, by presenting the findings for each subgroup of the equity stratifiers, we provide a benchmark for the government to identify where attention is much needed in the midst of limited resources. Finally, using the WHO’s HEAT software for the analysis confirms the reliability of the findings. Nonetheless, there are some limitations that need to be acknowledged. In this study, the focus was on the description of the nature of AFR inequality in light of the recommended dimensions of health inequality. We recommend the use of decomposition analysis to assess factors that could explain the disparities in AFR across various dimensions of inequality observed in this study. Again, the study used secondary data and so the authors had no influence over the selection and measurement of the variables. Additionally, there is a possibility of under-estimating AFR in the surveys due to pregnancy terminations among adolescents. There are other sociocultural variations that were not considered in this paper such as ethnicity and religious affiliation which could affect AFR. Therefore, future studies on AFR can examine the inequality patterns by ethnicity or religious affiliation.

## Conclusions

This study has revealed the existence of inequalities in AFR in Ghana to the advantage of adolescent girls in better socioeconomic classes and who are located in urban settings, with increasing disparity over the time period of the study. Policy makers need to institute measures to reverse the trends in the disparities by targeting subgroups which are highly disadvantaged. There is the need for strategies and interventions that targets these marginalized subgroups to address the disparities in AFR so as to effectively and efficiently deal with the challenges of high maternal and newborn morbidity and mortality associated with adolescent childbearing. Emphasis should be given to adolescents in rural areas and those in lower socio-economic class.

## Data Availability

The datasets generated and/or analyzed during the current study are available in the WHO’s HEAT version 3.1 [https://www.who.int/gho/health_equity/assessment_toolkit/en/].

## Abbreviations

AFR: adolescent fertility rate
D: Difference
EA: Enumeration Area
GDHS: Ghana Demographic and health Survey
HEAT: Health Equity Assessment Toolkit
LMICs: low- and middle-income countries
MMR: maternal mortality ratio
PAF: Population Attributable Fraction
PAR: Population Attributable Risk
PCA: Principal Component Analysis
PPS: Probability Proportional to Size
R: Ratio
SDG: Sustainable Development Goal
UI: Uncertainty Intervals
WHO: World Health Organization
